# Addressing Evaluation Barriers with Early Innovation Development for Adolescent-Focused Sexual and Reproductive Health Interventions

**DOI:** 10.1007/s11121-023-01578-2

**Published:** 2023-09-01

**Authors:** Kelly L. Wilson, Sarah Axelson, Whitney R. Garney, Kristen M. Garcia, Katy Suellentrop, Christi H. Esquivel

**Affiliations:** 1 Texas A&M University College Station, College Station, TX USA; 2 Power to Decide, Washington, DC USA; 3 RTI International, Washington, DC USA

**Keywords:** Innovation, Adolescent pregnancy prevention, Adolescent health, Evaluation challenges

## Abstract

Most evidence-based teen pregnancy prevention programs focus on individual-level sexual health outcomes (e.g., STIs, pregnancy, teen births). To expand program and intervention approaches within teen pregnancy prevention (TPP), the Department of Health and Human Services funded two grantees, Innovative Teen Pregnancy Prevention Programs (iTP3) and Innovation Next (IN) to support and enable early innovation to advance adolescent health and prevent teen pregnancy. The pipeline to support and enable innovation in adolescent health is complex, resulting in barriers and challenges to research and evaluation of novel programs. This paper presents some of the barriers encountered by the grantees. Data for this paper was collected from key personnel and secondary data sources. Focus group participants included seven representatives (*n* = 7) across the two organizations. Focus group questions assessed barriers related to innovative intervention development and evaluation. Key findings include four barriers to evaluation when fostering innovative adolescent-focused pregnancy prevention interventions. These included (a) funding constraints on evaluation activities, (b) innovation readiness for rigorous testing, (c) evaluation knowledge and expertise on innovation-development teams, and (d) challenges with evaluation requirements. Novel and promising system- and technology-focused interventions with the potential to impact TPP require alternative tools and approaches for evaluation. This would allow research to focus on how systems-level change mechanisms (i.e., policy, access to care) impact sexual risk behaviors and better understand ecological and social determinants of health for the priority population. The advancement of approaches to impact adolescent health identifies the need to expand the focus of evidence-based interventions beyond the adolescent themselves and understand approaches that impact external contexts and environments related to reducing sexual and reproductive health (SRH) risk-taking.

Most current evidence-based sexual and reproductive health (SRH) interventions for adolescents focus on modifying individual risk factors (e.g., condom use, contraception, substance use, violence) that can reduce the likelihood of unintended pregnancy or STIs (Griffin & Botvin, 2010; Grove et al., 2018). As a result, current measurement tools and evaluation methods for adolescent SRH interventions are grounded in performance measure data collection that focuses on individual health behaviors (Metz & Albers, 2014). By framing interventions and evaluations from an individual level, we ignore important socio-ecological factors.

Significant progress in the field of adolescent health is being made as program developers frame interventions from a social-ecological and systems approach. As program development models advance, evaluation methods must also adapt. Systems change interventions capitalize on the interactions between individuals and ecological factors that affect health outcomes. Examples of these approaches include increasing access to clinical services, improving linkages to wraparound services, and/or focusing on root causes of social inequities like school completion, early childhood education, or job training. Likewise, policy interventions can be effective in ensuring youth can meet their basic needs through various programs or technology-based approaches. However, these types of public health programs are excluded and not eligible for the evidence-based lists of programs that impact teen pregnancy and sexual health because they do not address individual-level outcomes (Mathematica, 2018). Since the programs or interventions do not exist in the evidence review, they are not adopted or eligible for federally funded replication opportunities.

The focus on individual outcomes puts restrictions on the types of evaluation approaches that public health practitioners can adopt because they are forced to prioritize sexual risk behavior outcomes, therefore, exclude systems-level initiatives focused on more distal change mechanisms, such as community-change, technology, or communication interventions (Morrissey et al., 1997; Waltz et al., 2019). Challenges exist for many innovative programs to be identified as evidence-based or having an impact because they require evaluation designs different from those widely accepted and used for individual-level approaches and outcomes (Nastasi & Hitchcock, 2009; Rigby et al., 2015). Interventions focused on social and ecological changes require system- or community-change measures. The purpose of this paper is to share the barriers and challenges related to innovation evaluation presented during an initiative to support and enable early innovation for adolescent-focused SRH interventions.

## Accelerating Early Innovation to Advance Adolescent Health Through Tier 2A Grants

The Department of Health and Human Services (HHS) funded a cohort of grantees for FY2015–FY2020, with two grantees supported under Tier 2A. The Tier 2A grantees served as intermediaries to support and enable early innovation to advance adolescent health and prevent teen pregnancy. Traditionally, funds to prevent teen pregnancy focused on the replication of evidence-based programs, effectiveness research, or rigorous evaluation of programs; therefore, an initiative for early innovation was new to the program.

To facilitate innovation, Texas A&M University’s Innovative Teen Pregnancy Prevention Programs (iTP3) was funded to focus on novel programs, whereas Power to Decide’s Innovation Next (IN) approached new technologies to advance adolescent health. Through the intermediaries, the HHS and Office of Population Affairs (OPA) built support to design innovative programs and technology initiatives. As the initiative unfolded, many factors influenced innovative development and, subsequently, the innovation’s likelihood of success.

### Innovation Intermediaries

iTP3 used a model of innovation (Garney et al., 2022), gained human insights, and encouraged co-design to initiate and accelerate program ideas which support adolescent SRH. System approaches included a focus on policy, environment changes, and community-level interventions. Beginning in 2015, three cohorts of teams were funded and supported to co-design innovative teen pregnancy prevention (TPP). Organizations applied for funding and proposals were selected based on the likelihood of success. Factors considered were the level of innovation, potential impact on adolescent health, and if the program could eventually be evaluated and scaled. To ensure that a variety of perspectives informed the development of innovations, innovative teams represented a range of topic areas, youth-serving professionals, and organizations (Garcia et al., 2022).

iTP3 funded more than 20 organizations over the 5-year period to engage in innovative program design. Program settings included health care systems, foster care, schools, community-based organizations, and homeless youth drop-in centers. Newly developed programs all intervened with priority populations with high risk for TPP and poor SRH outcomes.

Power to Decide created a unique incubator and accelerator program—Innovation Next—focused on technology-enabled ideas to prevent teen pregnancy. The goal of Innovation Next was to infuse design thinking within the teen pregnancy and adolescent SRH sector by teaching teams to apply design thinking to develop a new intervention and to improve their overall innovation capability. Beginning in 2015, Power to Decide funded twenty teams of innovators across three cohorts to solve complex challenges related to teen pregnancy prevention. Participants applied to the incubator in three-person, multi-disciplinary teams and were selected based on their response to an application that assessed openness to the design thinking process, diversity of skill set across the team, end-user audience, and potential for impact. To ensure that a variety of perspectives informed the development of innovations, applicants were encouraged to select “non-traditional” teammates from fields other than public health.

Each cohort culminated in pitch sessions on the innovations, and some teams received additional funding and guidance on developing, marketing, and refining their ideas and products to bring their innovation to market, to scale, or prepare for an impact evaluation. Aligned with the principles of design thinking, Power to Decide iterated and improved the incubator across cohorts based on lessons learned and feedback from the innovation teams. Universally, teams across all three cohorts expressed that Innovation Next changed how they approached their work—incorporating end-users’ voices, developing greater empathy for challenges adolescents face, and understanding the need to question their own approaches, think creatively about problems, and receive feedback as they develop solutions.

### Process to Drive Innovation

Both Innovation Next and iTP3 selected teams/organizations to participate in program development. The process varied between intermediaries but broadly consisted of a series of in-person workshops, customized coaching sessions, webinars, and virtual meetings. Teams simultaneously worked on their projects over a designated time period, focused on applying design thinking to the challenge they identified and receiving ongoing support from the intermediary staff.

During this process, teams were required to explore alternative solutions, rather than enter into the incubator with a pre-selected solution they may have had prior to support and funding. This allowed new ideas to emerge and frequently diverge from the initial idea. Teams’ success was ultimately influenced by their ability to embrace ambiguity and pivot. Innovation Next coaches were responsive to the specific needs of the teams, rather than following a scripted format or predetermined activities. The coaches provided a motivational push when teams were stuck, encouragement when the teams were feeling discouraged, and expertise when facing a challenge with the design thinking process. This customized support enabled teams to break through their barriers and challenge their own assumptions about what their respective priority audiences needed.

Both grantees were responsible for evaluation at the intermediary level during the 5-year project period. Intermediary evaluation included measures on planning and implementation of the innovation structures, outcomes of the innovation support process, and lessons learned. Neither of the two intermediaries pre-specified a required evaluation approach during the program development phase, rather provided tailored research support as needed. Evaluation support is most frequently focused on implementation and feasibility testing of new programs, to get programs ready for future testing. Both intermediary organizations and program developer teams were prohibited from rigorous evaluation per funding opportunity.

## Methods

To better understand the lessons learned from Tier 2A, a focus group explored the influences of success and barriers to innovation development and evaluation. The focus group was facilitated by an independent researcher to avoid bias (Greenbaum, 1998). Participants were all key personnel from the two intermediary organizations including the directors, evaluators, and lead programmatic staff (*n* = 7). Power to Decide is a not-for-profit organization, and Texas A&M is an academic university. Verbal consent was solicited and received from each participant prior to conducting and recording the focus group.

### Data Collection

The focus group took place at the end of the 5-year funding period. The focus group questionnaire was designed to capture lessons learned from intermediary organizations about their approaches and experiences to support and enable innovation in adolescent health. For this study, only questions and data pertaining to innovation evaluation were included in the analysis. The focus group occurred via Zoom and lasted approximately 120 min. Participant responses were audio-recorded and transcribed by an outside transcription firm. Once transcribed, the audio recording was erased to ensure confidentiality. All data collection was approved by the Texas A&M University Institutional Review Board prior to implementation.

Annual and semi-annual reports were made available by the two intermediary grantees. They were analyzed as a secondary data source and provided additional details to supplement the focus group results. Specifically, these reports provided performance measures for each organization that could not be captured during the focus group process.

### Data Analysis

Qualitative analysis was used to identify key themes from the focus group. Data were analyzed using grounded theory, an approach that begins without an a priori hypothesis or organizational framework. The analysis was exploratory; therefore, no coding scheme was pre-specified. The analysis was performed by four masters-level trained investigators (Bradley et al., 2007). Transcripts were coded independently, then codes were compared across investigators. In the event of a disagreement, the lead researcher resolved disputes (Patton, 1999). After reviewing and discussing the coding, 100% inter-rater reliability was achieved. Key themes were compiled by grouping like codes. The findings from the focus group were combined with metrics identified in the annual reports to add detail to contextual findings from the qualitative analysis.

## Results

The focus group analysis identified four key barriers and challenges to addressing evaluation while fostering innovative interventions in adolescent-focused SRH. These included (a) funding constraints for evaluation activities, (b) innovation readiness for rigorous testing, (c) evaluation knowledge and expertise on innovation teams, and (d) challenges with evaluation requirements and outcomes.

### Funding Constraints on Evaluation Activities

Per the funding opportunity, intermediaries were not allowed to use their budget to support rigorous evaluation for new interventions. The constraints were based on both intermediaries’ understanding of the funding opportunity and their notice of award. Therefore, both intermediary structures focused on early-stage innovation, not the evaluation of new programs. Throughout the 5-year grant cycle, the intermediaries realized they needed to be able to work with teams to feasibility test the new interventions, so they modified technical assistance to support these needs. However, most of the innovation teams were unable to collect outcome data needed to establish preliminary effectiveness; thus, they were not prepared to transition into Tier 2B funding mechanisms, rigorous testing, or impact evaluation opportunities. This challenge stalled further development and dissemination.

### Innovation Readiness for Rigorous Testing

One of the barriers experienced by innovation teams funded by both intermediaries is the amount of time it takes to move from innovation from early stages of development to a prototype that is ready for testing. All innovation teams moved at what, in the program development space, was considered a fast pace. However, there were necessary pivots and adjustments, as they moved through different stages of development, which took significant time.

Intermediary grantees created portfolios that showed success in fostering innovation development; however, they were challenged in bridging the gap from an idea to having the support data to support further evaluation. There was a significant need to continue the innovation pipeline by providing the necessary support and resources for evaluation as teams progressed finalized innovation design.

### Evaluation Knowledge and Expertise on Innovation Teams

Due to the nature of early innovation in adolescent SRH, innovation teams did not always have the expertise needed to support early evaluation and feasibility testing. Both intermediaries encouraged innovation teams to work with researchers and evaluators in various ways. Evaluators were both internal and external to organizations and helped teams gain insight from stakeholders, create evaluation plans, and assess intervention characteristics. The program and technology innovation teams required unique evaluation expertise due to their approach.

### Challenges with Evaluation Requirements and Outcomes

One of the challenges presented by the intermediaries was continuity in the innovation pipeline and providing the necessary support for enabling early innovation and resources to facilitate evaluation as teams progressed through the development process. Intermediary grantees created portfolios that showed their success to foster innovation; however, they were both challenged in bridging the gap from an idea to having the formative data to support readiness for an impact evaluation.

Innovations addressed non-traditional outcomes. For example, technology-focused interventions focused on outcomes from the communication discipline, which assess elements across technology platforms, structures, and communication channels (MacDonell & Prinz, 2017). Another example is a programmatic intervention that increased advocacy for adolescent health, which focused on alternative outcomes and protective factors like youth engagement and skill building. Other community-level outcomes targeted include relationships, networks, policies, environment change, or system structures. The intermediaries worked with teams to iterate, deviate, and be flexible in creating these novel products through system-level programs or technology platforms that were meaningful to stakeholders and had the potential to impact their sexual and reproductive health. At the time of design and development, much of the evaluation focused on formative evaluation. Thus, the time and funding needed to accomplish this created a huge gap between the Tier 2A portfolios and the readiness for those prototypes to be considered ready for rigorous testing and impact evaluation.

## Discussion

As part of their initial work plan, both intermediaries cycled in “new” innovation teams throughout the 5-year grant cycle. This approach kept the innovation ideas fresh and iterative while allowing innovation teams to work along a continuum of innovation development. However, the approaches from both intermediaries were more focused on design than evaluation, which impeded the ability to provide evaluation support to prepare early-stage ideas to apply for rigorous evaluation either as the innovation team or intermediary-level funding was ending. There was little focus on feasibility or pilot data that constrained early findings of many of the interventions developed from the initiative. In considering the bridge between early innovation and interventions ready for rigorous testing, there is a gap related to evaluation needs and readiness. This gap was primarily caused by the focus on early development using insight data and restrictions on securing data that would have been acceptable for an impact or effectiveness study.

Sponsors, funders, and intermediaries may consider the various mechanisms that drive early innovation as well as the types of innovations they hope result from an initiative to create and support new ideas. In this case, intermediaries recognized mechanisms as space, process, and partnerships, and this was successful in fostering innovative ideas. OPA separated the intermediaries to focus on programmatic and technology-focused innovations. Yet, it was discovered that additional framing around innovation and the role of evaluation could have supported the intermediaries to address the innovation product and readiness for evaluation. For example, both intermediaries learned through trial and error about team makeup. If intermediaries were to include evaluation support or teams were to include an evaluator, then evaluation may have been considered at the onset or in the early stages of development.

Involving an evaluator or having an evaluation lens at the onset of innovation will allow for research to be conducted while developing a novel prototype and including evaluation considerations. There are various methods that can be used to evaluate unique programs and technologies that use evaluation methods other than a randomized controlled trial or quasi-experimental impact study. From the intermediaries’ perspectives, a more flexible evaluation approach allows for outcomes to be measured in diverse and context-appropriate ways. For example, using an implementation science approach allows researchers and evaluators to better understand the full aspect of a program and seeks to answer evaluation-related questions beyond individual outcomes (Ghate, 2015). Based on the programmatic or intervention goals, a “one size fits all” approach is neither appropriate nor helpful (Bauer & Kirchner, 2020).

The intermediaries felt they were able to support and foster innovation. However, there is still more to learn about how to encourage the innovation teams to “innovate,” yet give them a launching pad to move beyond feasibility testing to collecting pilot test data. There was a small percentage of teams that participated in the innovation pipeline that had the type of data needed to support rigorous testing and impact evaluation and future consideration to be named an evidence-based intervention. These teams had unique aspects that set them apart from other innovation teams which allowed for collecting feasibility or early pilot test data. Examples include evaluation capacity on the team or from an outside source working on their team or project and supplemental funding beyond the intermediaries that allowed for further development and research on the innovation.

## Conclusions

Priorities for this federal initiative included developing and fostering innovation for adolescent-focused sexual and reproductive health interventions in programmatic or technology areas. Innovation teams were supported by the intermediaries, iTP3 and IN, throughout the development process; however, most teams did not focus on developing or carrying out a formal evaluation plan for their early-stage innovation. This lack of focus on evaluation was intentional, as most programs were not ready to develop an evaluation component, and the intermediaries were not offered fiscal resources to support the evaluation of the innovations. Additionally, there were OPA grantees funded under a different tier of funding (Tier 2B) whose organizations were awarded funds to conduct a rigorous evaluation of a fully developed, feasible idea. If the innovation-focused intermediaries focused evaluation efforts on these innovations, this may have created redundancy in other tiers of funding. Additionally, because this was a first-time initiative from OPA, understanding more about the development of innovations for adolescent health interventions was important. Therefore, funders and sponsors of innovation and early development may consider the lessons learned from this initiative compared to what is known about rigorous testing and impact evaluation challenges and barriers.

The process to support and enable innovation and trailblazing interventions in adolescent sexual and reproductive health is novel, yet complex. Much of the literature related to teen and adolescent pregnancy prevention programs focuses on the rigorous testing and review which lead to the identification of evidence-based programs (McLeroy et al., 2016). The outputs of these program evaluations focus on individual-level variables and performance measures, which is typical in public health and prevention. Yet, these evaluations disregard population health and community-level or organizational-level aspects of an intervention (Coyle & Glassman, 2016, Garney et al., 2019). To understand system-level and technological approaches, evaluation efforts need to take into consideration and allow outcomes and change mechanisms beyond individual and individual-level behaviors. This approach allows implementation and evaluation teams to carry out multi-level evaluations that provide insight into intra-personal, organizational, and community-level change and impact. This innovation initiative, supported by OPA, highlights the promise of technology and system-focused interventions to advance teen pregnancy prevention.

## Implications

Both intermediary grantees were able to evaluate the processes and strategies they incorporated throughout the duration of their project and identify the impact they had on supporting and enabling early innovation for adolescent health. However, both intermediaries experienced barriers and challenges in supporting evaluation at the innovator level because of traditional evaluation requirements. Recommendations include the consideration of optimal evaluation methods and frameworks for collecting outcomes based on the focus of the innovation and research questions. Evaluation metrics for adolescent health programs and interventions tend to be constrained to individual-level outputs, causing a gap in evaluation data for system-level outputs (i.e., community, organizational, and policy-level outputs). By allowing additional options for evaluation methods and metrics, the evidence base in adolescent health can be expanded to understand how early innovation and novel approaches impact technology, community, and systems-focused outcomes.

Through this initiative, intermediaries were able to break down most obstacles to innovation for the communities and teams they were working with. Innovation teams were able to address complex issues within adolescent health and fundamentally challenge systems and communities to impact social and environmental dimensions. Teams were motivated, incentivized to be innovative, and they embraced the innovation process and innovation success received accolades from key stakeholders. However, as innovations continued and expanded, there was a gap in readiness to evaluate the innovation’s effectiveness. In short, innovation teams were adapting to new conditions and contexts, and they desired knowledge about the likeability and adoptability of the interventions. Because teams were developing an early innovation, the real-time solutions and interventions were not to the point where they were ready for traditional formative and summative evaluation (Patton, 2011).
